# Perineural Invasion, Pain and Immunosuppression Across Solid Tumours

**DOI:** 10.3390/curroncol33070434

**Published:** 2026-07-20

**Authors:** Przemysław Dybcio, Anna Kuraś, Mikołaj Dyrka, Michał Iwaszko, Joanna Pec, Jakub Kleinrok, Agnieszka Korolczuk

**Affiliations:** Department of Clinical Pathomorphology, Medical University of Lublin, Kazimierza Jaczewskiego 8b, 20-090 Lublin, Poland; dybcio.przemek@wp.pl (P.D.); aniakuras28@gmail.com (A.K.); mikolajdyrka@gmail.com (M.D.); iwaszkomax@gmail.com (M.I.); klejs.90@gmail.com (J.K.)

**Keywords:** perineural invasion, perineural niche, neuropathic pain, neuroinflammation, head and neck cancer, immunosuppression, cancer–nerve crosstalk

## Abstract

Perineural invasion (PNI) is a distinct route of cancer spread associated with neuropathic pain, local recurrence, and poor survival across many solid tumours. Increasing evidence shows that PNI is not only a structural pattern of invasion but also a dynamic biological process involving neurodegeneration, nociceptor sensitisation, and marked local immunosuppression. This narrative review synthesises experimental, translational, and clinical data on the molecular, neurological, and immunological mechanisms of PNI in solid malignancies. PNI arises through complex crosstalk between tumour cells, Schwann cells, macrophages, fibroblasts, and neurotrophic pathways, leading to peripheral nerve remodelling, axonal degeneration, and abnormal regeneration. These changes promote neuropathic pain through ion-channel dysregulation, neurotrophin-driven sensitisation, and pathological neuroplasticity. At the same time, PNI creates an immunosuppressive microenvironment enriched in Tregs, M2 macrophages, and myeloid-derived suppressor cells, shaped by cholinergic, adrenergic, and neuropeptidergic signalling, which may contribute to immune exclusion and resistance to immunotherapy. We propose that PNI should be understood as a neuro-immuno-metabolic process and that recognising the PNI–pain–immunosuppression triad may support the development of targeted neuroprotective, analgesic, and immunomodulatory therapies.

## Simple Summary

Perineural invasion is a pattern of tumour cells spread along or within peripheral nerves. It occurs in many solid tumours and is associated with neuropathic pain, local recurrence, and poorer prognosis. This review summarises the current state of knowledge indicating that perineural invasion is not only a structural pattern of tumour growth but also an active biological process involving nerve damage, abnormal nerve remodelling, pain sensitisation, and local attenuation of the antitumour immune response. These changes are driven by communication between tumour cells, nerves, Schwann cells, macrophages, fibroblasts, and neurotrophic signalling pathways. We propose that perineural invasion, pain, and immunosuppression be viewed as a related neuroimmunological process. Recognising this relationship may inform future research and aid in the development of therapies aimed at protecting nerves, alleviating cancer-related pain, and modulating the immune response.

## 1. Introduction

### 1.1. Clinical Relevance of Perineural Invasion

Perineural invasion (PNI) is a pathological process regarded as an additional form of extension in solid tumours, characterized by tumour-cell infiltration of peripheral nerves. PNI is a histopathological finding and should be distinguished from perineural tumour spread (PNTS), which refers to the macroscopic extension of a tumour along nerves detectable by imaging modalities, particularly magnetic resonance imaging (MRI). Unlike PNI, PNTS represents a more advanced stage of perineural involvement and is associated with a poorer prognosis, more extensive disease, and more pronounced neurological manifestations [1].

PNI was initially described by Cruveilhier in 1835 during investigations of breast cancer cells, in which tumour spread along neural pathways was observed to facilitate extension into the intracranial fossa [2]. Over recent years, extensive research on this phenomenon has confirmed that PNI also occurs in other malignant solid tumours, including pancreatic, prostate, colorectal, gastric, and head and neck cancers. Head and neck cancers, as well as pancreatic cancer, exhibit a particularly high propensity for perineural invasion. The incidence of PNI varies depending on the tumour grade, histological subtype, and anatomical location. The presence of PNI is typically associated with worse prognosis, worsening clinical symptoms, and a reduced overall survival rate [3]. The definition of the PNI process has evolved over the years. According to the currently accepted definition proposed by Liebig et al. based on the original criteria described by Bockman et al., perineural invasion is diagnosed when a nerve is in close proximity to the tumour and neoplastic infiltration involves at least 33% of the nerve circumference or when tumour cells are identified within any of the layers constituting the nerve sheath.

### 1.2. From Nerve Tropism to the PNI–Pain–Immunosuppression Triad

As an introductory clarification, it is necessary to define the concept of the perineural niche, which will be frequently referenced throughout this study. This term refers to tumour–nerve crosstalk and denotes a specialised communication network composed of a variety of soluble signalling molecules and their corresponding receptors, such as the NGF-, TGF-, and MMP-family molecules described extensively later in the paper. This network enables indirect communication between neoplastic cells and adjacent, non-invaded neural cells. The reciprocal interactions between tumour cells and neural cells through the secretion of specific chemoattractants or the activation of specific signalling pathways, leading to the directed migration of cancer cells toward nerves, are referred to as neurotropism, which constitutes the fundamental mechanism initiating the subsequent process of perineural invasion. Through this bidirectional molecular signalling, cells are able to respond to one another by activating or suppressing specific signalling pathways [4].

Perineural invasion also appears to have a direct impact on the development of neuropathic pain and nerve dysfunction, which represent particularly distressing symptoms for patients [5].

### 1.3. Scope and Aims of the Review

In this article, we aim to summarize the current state of knowledge regarding the process of PNI and to propose a novel hypothesis describing a functional triad of PNI–pain–immunosuppression observed in oncologic patients, especially those diagnosed with pancreatic cancer or head and neck cancer with the presence of PNI. Our objective was to develop a narrative review, with a particular focus on a hypothesis-driven framework.

Furthermore, by comparing the course and biological characteristics of PNI across malignancies of different organ systems, we seek to identify shared patterns, as well as tumour-specific features, and to discuss their therapeutic implications. We also aim to highlight existing gaps in knowledge and outline emerging directions for future research on this phenomenon.

### 1.4. Methodology

We identified articles published between January 2009 and March 2026. In addition, we included a seminal publication from 1862, identified through the reference list of one of the retrieved articles. The inclusion of this historical report was intended to highlight how early the phenomenon of perineural invasion (PNI) was recognised and described, as well as to illustrate the evolution of our understanding of this process over time. A targeted literature review was conducted using PubMed, Scopus, Google Scholar, and Web of Science.

The primary objective of this review was to provide a comprehensive overview of PNI, with particular emphasis on its role in cancer-associated pain, immunosuppression, and tumour dissemination along neural structures. The literature search was performed using the following combinations of keywords: “perineural invasion”, “perineural niche”, “head and neck cancer”, “immunosuppression”, “pancreatic cancer”, “breast cancer”, “prostate cancer”, “colorectal cancer”, “gastric cancer”, “neuroinflammation”, “cancer–nerve crosstalk”, “tumour microenvironment”, and “pain”.

The study selection process was performed in a structured but non-systematic manner, consistent with the narrative character of the review. First, the titles and abstracts were screened for relevance to perineural invasion or to biological mechanisms that could further elucidate this process. Subsequently, the full texts of potentially eligible articles were assessed, with priority given to clinical trials, meta-analyses, observational studies, and guideline-based publications. Additional relevant articles were identified through manual screening of reference lists of key publications.

Publications were excluded if they were not relevant to the scope of the review, did not address PNI-related mechanisms or clinical implications, or focused exclusively on aspects of perineural invasion outside the thematic focus of this article. Because this was a narrative review, the evidence was synthesised qualitatively, and no formal systematic review protocol, PRISMA-based study selection procedure, or risk-of-bias assessment was performed. Publications were included if they addressedthe potential association of perineural invasion with pain and immunosuppression, the development and clinical manifestations of PNI, selected malignancies, targeted therapeutic strategies, or relevant molecular and cellular mechanisms.

## 2. Biology of Perineural Invasion and the Perineural Niche

### 2.1. PNI Patterns in Cancers

PNI is a common feature of many solid tumours, but reported incidence rates vary significantly depending on the malignancy type, diagnostic criteria, and detection sensitivity. It is especially prominent in pancreatic ductal adenocarcinoma (PDAC), gastric carcinoma, prostate cancer, head and neck squamous cell carcinoma (HNSCC), colorectal cancer, and cervical cancer [4,6]. Statistical discrepancies often stem from a lack of uniform reporting standards and variations in staining techniques. In particular, the use of immunohistochemistry (IHC) for markers such as S-100 or PGP9.5 substantially increases PNI detection compared with conventional haematoxylin and eosin (H&E) staging [7,8].

PDAC has one of the highest frequencies across all malignancies. Studies indicate a range of 70% to 100% of cases. Some analyses narrow this range to 71–98% or 80–100%. It is often present in the absence of vascular or lymphatic spread and independently predicts worse survival and recurrence [6,9]. It is a main cause of severe neuropathic abdominal and back pain [3].

In prostate cancer, the incidence of PNI is very high, reported in the range of 75–100% or 85–100% [10,11]. However, a meta-analysis of more than 13,412 patients showed a much wider range of 12.4% to 83.6% [4]. The key mechanism for the spread of cancer beyond the prostate capsule (extraprostatic extension) is nerve invasion [12]. It is associated with a higher risk of recurrence [10].

PNI in HNSCC occurs in approximately 5.2% to 90% of patients [4]. In squamous cell carcinoma of the head and neck mucosa, the range is typically 25–80% [11]. Specifically for oral squamous cell carcinoma (OSCC) and squamous cell carcinoma (SCC), the incidence of nerve invasion reaches 80% [10,11,13]. A characteristic pattern here is invasion of the facial nerve, leading to paralysis of the facial muscles, causing persistent facial pain [5].

The incidence of PNI in colorectal cancer (CRC) ranges from 9% to 38.9% or 15.7% to 38.9% [4,7]. The detection method has a key impact on the statistics: routine HE staining shows PNI in approximately 18.5% of patients, while the use of immunohistochemistry (S-100 protein) increases this rate to 67.3% in the same study group [8]. The frequency increases with stage: from 10% in stage I–II to 40% in stage IV. PNI in CRC is considered a strong marker of early metastasis and an independent factor shortening overall survival [7].

When it comes to gastric cancer, PNI is found in 6.8% to 75.6% of cases [4]. Other studies report a range of 31.7–65%, with the presence of infiltration always correlating with a poorer prognosis and a more invasive phenotype [12]. Similarly, in bile duct cancer, where the incidence of PNI ranges from 56% to 88%, neural invasion reflects highly aggressive tumour biology [4,12].

PNI is also an important prognostic factor in cervical cancer (7–41.7%) [8] and in breast cancer (approximately 33%), where it correlates with advanced clinical stage and lymph-node metastasis [10]. In advanced disease, PNI may contribute to chronic neurological manifestations, including neuropathic pain, paresthesia, and sensory loss [14].

### 2.2. Perineural Niche: Cellular Components and Signalling Networks Driving Cancer–Nerve Crosstalk

The perineural niche is a unique and dynamic microenvironment in which there is intensive and bidirectional communication between cancer cells and elements of the nervous system [15]. This process, known as PNI, involves more than just passive movement of cancer cells along low-resistance pathways; it also involves active remodelling of tissues driven by specific molecular signals [7,11,15].

Peripheral nerves are protected by three layers of connective tissue that, together, form an effective barrier against mechanical injury and the spread of infection. The first layer is the epineurium. This layer consists of collagen and elastin and also includes blood and lymphatic vessels. The second layer is the perineurium, and it is the middle layer. It consists of several layers of fibroblasts and a basement membrane. This layer functions as a selective barrier, helping to regulate the internal environment of the nerve fascicles. The innermost layer, the endoneurium, surrounds individual axons, along with their associated Schwann cells, offering delicate structural support and maintaining the microenvironment necessary for proper nerve function [11,15]. PNI is defined as the presence of tumour cells in any of these layers or their adhesion to the nerve of an area covering at least 33% of its circumference. Many types of cells work together in the perineural niche to create an environment conducive to tumour progression [5,7,11].

Schwann cells play a central role in this process. Under the influence of tumour-derived signals, they dedifferentiate into so-called reparative Schwann cells (RSCs) [11]. These cells form tumour-activated Schwann-cell tracks (TASTs), which are synchronised cellular trajectories that physically intertwine with and guide cancer cells along nerve fibres, facilitating their directional migration [7,16]. Macrophages also contribute significantly to the perineural niche. Endoneurial macrophages are recruited by colony-stimulating factor 1 (CSF-1) secreted by tumour cells [11,17]. In response, these tumour-associated macrophages (TAMs) secrete glial-cell-line-derived neurotrophic factor (GDNF), a potent chemoattractant that enhances the migration of cancer cells toward nerves [4,11,17]. Cancer-associated fibroblasts (CAFs) further support perineural invasion by remodelling the extracellular matrix (ECM). They secrete matrix metalloproteinases, particularly MMP-2 and MMP-9, which degrade ECM components and facilitate the physical invasion of tumour cells into nerve structures [3,7,11]. This dynamic cellular interplay within the perineural niche is fuelled by localised metabolic alterations such as hypoxia and the Warburg effect, which lead to the accumulation of lactate and the upregulation of neurotrophin pathways like NGF/TrkA [3,10,11].

### 2.3. Shared Molecular Pathways in PNI and Immunosuppression

Both perineural infiltration and immune suppression are closely linked through bidirectional molecular communication, forming a neuroimmune feedback loop [3,10]. Signals from nerves, stromal cells, and tumour cells promote tumour-cell migration towards neural structures while simultaneously limiting effective antitumour immune responses. These mechanisms increase the invasiveness of the tumour cells and create an immunosuppressive microenvironment around the infiltrating nerves [3,8].

Common signalling pathways include the CXCL12/CXCR4 axis, in which CXCL12 secreted by the stroma and nerves attracts tumour cells to nerve structures while limiting T-cell infiltration within the tumour [3]. Transforming growth factor beta (TGF-β) enhances the invasiveness of tumour cells and the EMT process in PNI [3,8] while simultaneously inhibiting the cytotoxic functions of T and NK cells and promoting the activation of Treg lymphocytes and the polarisation of macrophages towards M2 [3]. Neurotrophins, including NGF, via TrkA and TrkB receptors, support chemotaxis between the nerve and the tumour and modulate neurogenic inflammation and T-cell responses [3].

Perineural invasion is sustained not only by pro-migratory signals but also molecules that shift the local immune balance toward suppression. Neurotransmitters and cytokines such as acetylcholine, norepinephrine, TGF-β, and IL-6, together with checkpoint pathways like PD-L1/PD-1, reduce cytotoxic T-cell and NK-cell activity while promoting the accumulation of immunosuppressive populations including Treg cells, M2, and myeloid-derived suppressor cells [7,8,18,19,20].

As a result, nerves not only enable the physical expansion of the tumour but also create an immunological ‘shield’, facilitating the escape of cancer cells from the supervision of the immune system [18,21].

### 2.4. Perineural Invasion as a “Neurodegenerative-like” Process

Emerging evidence indicates that invaded nerves in PNI undergo pathological changes resembling those seen in neurodegenerative disorders. Recent research on mouse models has redefined the role of PNI as an active neurodegenerative-like process rather than a passive route of expansion. The nerves involved in PNI express the fundamental pathological features typical of classical neurodegenerative disorders, including neuroinflammation, mitochondrial dysfunction, and disrupted cellular metabolism, supporting this shift in perspective [21].

The study of the transcriptomic landscape of PNI, as revealed by RNA sequencing, shows a high degree of similarity with neurodegenerative diseases of the central nervous system, as seen in Alzheimer’s, Parkinson’s, Huntington’s, and ALS. Furthermore, nerves involved in PNI exhibit dysregulation of genes closely associated with neurodegeneration, such as *Trem2*, *Ch25h*, *Reln*, and *Nos1*. This provides additional support for the molecular similarity between these conditions [21].

At the cellular and molecular levels, this active neurodegenerative process is characterised by structural disorganisation, mitochondrial dysfunction, and robust neuroinflammation within the nerve microenvironment. Cancer-cell infiltration and mechanical compression disrupt the nerve architecture, causing myelin separation; thinning; and downregulation of key myelination genes, including *Mag* and *Mbp*. These changes are accompanied by infiltration of CD4^+^ and CD8^+^ T cells, F4/80^+^ macrophages, and intraneural CD68^+^ phagocytic cells, which colocalise with axonal (NF200) and myelin (MPZ) markers, indicating active clearance of damaged axons and myelin reminiscent of Wallerian degeneration. Concurrently, accumulation of abnormal mitochondria and dysregulation of genes involved in oxidative phosphorylation and ATP production result in mitochondrial dysfunction and impaired cellular metabolism [21].

Although PNI induces markers of nerve repair and plasticity, such as the upregulation of GAP43 and nestin, the tumour environment forces the nerve into a state of continuous, aberrant remodelling. As a result, normal resolution mechanisms fail, and the nerve remains trapped in a maladaptive regenerative state [3,6]. In terms of function, these degenerative changes result in a considerable reduction in mechanosensitive afferents, as well as changes in conduction velocity, which clinically manifests as refractory neuropathic pain, paraesthesias, and motor deficits, especially with malignancies such as head/neck cancers and pancreatic carcinoma [5,21].

In summary, the extant evidence clearly shows that nerve injury causes a self-perpetuating, feed-forward cycle. In this loop, cancer-cell infiltration and nerve compression induce oxidative stress and the release of inflammatory cytokines, thereby amplifying neural injury beyond the body’s capacity for repair [3,21]. Recognising the similarities between PNI and neurodegenerative diseases could lead to new opportunities for the repurposing of drugs and the development of neuroprotective strategies to mitigate cancer-associated neuropathies [21,22].

## 3. Perineural Invasion and Cancer-Induced Pain

### 3.1. Structural Nerve Injury and Demyelination in Perineural Invasion

During PNI, neural damage occurs through both direct mechanical compression by tumour cells and indirect microenvironmental mechanisms, such as neuritis and pathological nerve plasticity [19,23]. Structural injury and algogenic mediators from the tumour microenvironment (TME) activate pain receptors on primary afferent neurons, enhancing pain transmission to the spinal cord [12,23].

Degeneration of both myelinated and unmyelinated fibres is observed in PNI. There is a significant reduction in the density of these nerve fibres, demyelination, and enlargement of Remak bundles [12]. In experimental models, such as sciatic nerve injection with oral squamous carcinoma cells, increased demyelination, axonal disorganisation, spontaneous pain, and mechanical nociception have been demonstrated [12,23]. Tumour-induced sensory nerve injury is associated with the upregulation of activating transcription factor 3 (ATF-3), hypertrophy of satellite cells in the dorsal root ganglion (DRG), and activation of microglia in the dorsal horn of the spinal cord [23].

With the progression of PNI, maladaptive plasticity triggers a loss of sympathetic fibres, nerve hypertrophy, and neuroinflammation [23].

### 3.2. Ionic Channel Remodelling in PNI-Associated Pain

During tumour–nerve interactions, the upregulation of voltage-gated sodium channels (Nav) in injured sensory neurons lowers the activation threshold, promoting abnormal discharges and amplifying sensory signalling to the spinal cord. This process facilitates the transition from acute to long-lasting neuropathic pain [12]. Moreover, the activation of TrkA receptors by NGF in the tumour environment is directly linked to the increased expression of Nav1.8 and Nav1.9. These channels, which are preferentially expressed in nociceptive neurons, play a key role in the development and maintenance of chronic pain [12,13].

TRP channels, including TRPV1 (vanilloid type 1) and TRPA1 (ankyrin type 1), serve as the initial transducers of environmental stimuli into nociceptive signals within the peripheral nervous system (PNS) microenvironment. Significant upregulation of TRPV1 is evident in tumour-innervating dorsal root ganglia (DRGs), correlating with increased neurite extension toward cancer cells and amplified pain perception. While TRPA1 is persistently sensitised by oxidative stress within the TME, elevated TRPV1 expression directly correlates with higher clinical pain scores and increased tumour-directed neurite extension [12,23]. This channel remodelling facilitates the peripheral release of algogenic neuropeptides, establishing a sustained state of peripheral sensitisation often resistant to conventional analgesics [12,13,16,23].

### 3.3. Roles of NGF, GDNF, NPY, Substance P, and CGRP in Nociceptor Sensitisation and Tumour Innervation

The neurotrophic and neuropeptidergic environment within the perineural niche acts as a bidirectional catalyst for tumour expansion and nociceptor sensitisation [12,13,16].

Nerve growth factor (NGF) binds to the high-affinity tropomyosin receptor kinase A (TrkA) and p75 neurotrophin receptor (NGFR/p75^NTR) on sensory neurons and tumour cells [12,13,16]. NGF signalling promotes axonogenesis, driving the sprouting of nociceptive fibres into the tumour microenvironment, increasing local nerve density but also sensitising nociceptors. Receptor pathways mediate distinct facets of pain, since TrkA activation primarily drives thermal hyperalgesia, while p75NTR mediates mechanical allodynia [13]. Activation of the NGF/TrkA pathway promotes tumour-cell proliferation, invasion, and metabolic reprogramming, e.g., via the Warburg effect, correlating with increased PNI severity [5,13].

GDNF functions to supplement the role of NGF by activating the RET receptor complex, driving neurite outgrowth toward the tumour and sustaining supportive Schwann cells, thereby further enhancing tumour innervation and progression [6,19].

Neuropeptides including neuropeptide Y (NPY), substance P (SP), and calcitonin gene-related peptide (CGRP) are released from sensory neurons infiltrating tumours and from tumour-associated glial cells. By acting on G-protein-coupled receptors on nociceptive neurons, they induce depolarisation and lower the action-potential threshold, driving persistent pain signalling [19]. SP activates neurokinin-1 (NK1) receptors, thereby facilitating excitatory signalling and local neurogenic inflammation. CGRP amplifies this inflammatory response and increases vascular permeability, which may result in the recruitment of immune cells capable of secreting neurotrophic factors [19,23]. It also exerts potent immunomodulatory effects, including downregulation of antitumour immune responses through the depletion of CD8^+^ T cells and reductions in NK recruitment and cytotoxicity [16,23]. NPY modulates both excitatory and inhibitory signalling in nociceptors by acting via Y1 and Y2 receptors, contributing to chronic pain states [16].

Collectively, NGF, GDNF, NPY, SP, and CGRP form a feed-forward loop in which increased innervation promotes tumour progression and metastasis through perineural invasion and heightens persistent pain transmission. As such, it has been proposed that these mechanisms can be targeted for therapeutic purposes in a manner that will limit cancer spread and provide cancer-pain relief. This has been demonstrated in preclinical models for NGF, RET, and neuropeptide receptors [6,13,16,19].

### 3.4. Transition from Acute to Chronic Neuropathic Pain

In cancer patients, the transition from acute pain to chronic neuropathic pain is a dynamic process reflecting permanent peripheral nerve damage, pathological neuroplasticity, and progressive central sensitisation [23]. Tissue inflammation, tumour expansion, and surgical trauma are usually linked to acute oncological pain. In contrast, chronic pain develops when persistent nociceptor stimulation induces lasting structural and functional alterations within the nervous system [19].

Ectopic discharges from remodelled ion channels fuel this transformation, driving functional and structural reorganisation within the spinal cord and higher cortical structures, such as the anterior cingulate cortex and thalamus [13,19,23].

In PDAC, one of the most painful malignancies, perineural invasion is closely associated with neuropathic pain [3,17,18,23]. As the disease progresses, axonal degeneration, demyelination, and nerve hypertrophy develop, clinically presenting as persistent abdominal and back pain that is often resistant to standard opioid therapy [23].

Cancer surgery may also initiate persistent postoperative neuropathic pain (PPSP), particularly following procedures such as mastectomy or thoracotomy [12,22]. Intraoperative nerve cutting or stretching induces abnormal Schwann-cell plasticity, leading to the formation of TASTs, which facilitate tumour invasion and intensify ectopic neuronal discharges [22,23]. Additionally, chemotherapy-induced peripheral neuropathy (CIPN), caused by mitochondrial damage and axonal degeneration, frequently overlaps with cancer-related pain, reinforcing a self-perpetuating cycle of chronic neuropathic pain [22].

### 3.5. Determinants of Pain Variability in Perineural Invasion

PNI exhibits distinct clinical differences, since in certain malignancies, it is the primary source of debilitating neuropathic pain, while in others, it remains clinically “silent”, acting primarily as a conduit for expansion without early sensory symptoms [7,12,23].

Pain-dominant PNI, such as in pancreatic and head and neck cancers, is almost inseparable from severe sensory dysfunction [12,21,23,24]. PNI is a hallmark of PDAC. The celiac plexus is often involved, leading to severe abdominal and back pain in approximately 80% of patients. This pain results from pancreatic neuritis, massive neural hypertrophy, and increased nerve density, collectively termed PDA-associated neural remodelling (PANR) [3,17,18,23,24]. Due to the dense sensory innervation of the maxillofacial region, PNI in HNSCC manifests as intractable facial pain, burning sensations, or numbness. Invasion of the trigeminal nerve (CN V) and facial nerve (CN VII) specifically causes persistent pain and progressive paralysis [12,21,23].

The “silent” or less painful PNI appears in prostate and colorectal cancers [7,11,12,23]. Despite high histological prevalence, PNI in these organs often lacks early painful manifestations. In prostate cancer, PNI primarily serves as a mechanism for extraprostatic extension. Pain is often absent in early stages, typically only appearing in advanced disease if the tumour invades the lumbosacral plexus [11,12]. In colorectal cancer, even though the colon is highly innervated, PNI is often underreported and less frequently associated with early clinical pain compared to PDAC [7,23].

The clinical difference in pain perception is likely driven by four microenvironmental factors: the type and density of innervation, biological affinity and neurotrophins, local TME and neural plasticity, and suppression of pain signals [7,12,21,23].

Painful PNI (pancreas and HNSCC) occurs in regions dominated by nociceptive sensory fibres. In contrast, prostate PNI often involves interactions with autonomic (sympathetic/parasympathetic) fibres, which support tumour growth via neurotransmitters like noradrenaline but do not directly transmit pain signals in the same capacity as nociceptors [10,12,22].

PDAC cells show high expression of neurotrophins like NGF and BDNF, which create a strong chemotactic gradient and induce pathological axonogenesis [16,24]. In CRC, the expression of these neurotrophins and their receptors is significantly lower, leading to a lack of significant biologic affinity between cancer cells and neurons [7].

Painful PNI is characterized by aggressive neural remodelling and endoneurial inflammation [3,12]. CRC and early prostate PNI often lack this remarkable neuroplasticity, allowing the tumour to “hijack” nerves as “channels” without triggering a robust inflammatory pain response [7,23].

Cunning tumours may work with surrounding TME cells to suppress early pain signals. For instance, Schwann cells in early PDAC can secrete factors like CXCL12 or IL-6 that may initially inhibit pain, delaying early detection until the injury is severe [12,23].

## 4. Perineural Invasion and the Immunosuppressive Tumour Microenvironment

### 4.1. Immune-Cell Composition Around Invaded Nerves

In the PNI microenvironment, Tregs play an important role in the development of immunosuppressive activities. Tregs are generated from naive CD4^+^ T cells by different TME cells, such as transforming growth factor-beta (TGF-β) and interleukin-10 (IL-10), resulting in the differentiation of FOXP3^+^ Tregs. The Tregs then produce IL-10, TGF-β, and IL-35, which inhibit the proliferative and cytotoxic activities of CD8^+^ CTLs and NK cells. Tregs are also responsible for the suppression of cellular metabolism by the production of adenosine via the action of ectonucleotidases CD39 and CD73. They are also responsible for the suppression of the maturation of DCs and antigen presentation, thereby limiting secondary immune-response generation against tumour cells infiltrating nerves, thereby limiting immune surveillance in the perineural space [20].

M2 macrophages, which are abundant in the TME, promote tumour progression by promoting angiogenesis and immunosuppression. The hypoxic environment caused by high lactate levels enhances the secretion of IL-6, TNF, CCL5, and CCL1 by M2 macrophages. M2 macrophages are also responsible for the suppression of T-cell activation via the expression of PD-L1 [18].

MDSCs are a heterogeneous group of immature myeloid cells that exhibit potent immunosuppressive activities. MDSCs, located within the TME, play a role in immunosuppression by inhibiting effector lymphocytes, such as CD8^+^ T cells, thereby promoting an immunosuppressive type of inflammatory response, which supports tumour progression. Neural communication, especially in the cholinergic system, has been shown to modulate the composition of tumour-infiltrating cells, especially MDSCs. Neuroimmune communication is critical in the development of PNI through the recruitment of myeloid cells into neural structures, thereby promoting the migration and invasion of tumour cells along nerves [25].

Mast cells, recruited by mediators from tumour and stromal cells, activate and degranulate within the perineural microenvironment, releasing different bioactive mediators. These include regulatory cytokines and proteolytic enzymes, which act on the tumour microenvironment. The immunomodulatory role of mast cells, via interactions with different immune cells, leads to the development of a microenvironment that lacks antitumour activities. The proteolytic enzymes released by mast cells act on the extracellular matrix, thereby remodelling the extracellular matrix and promoting the disorganisation of the tumour stroma within the nerve sheath, thereby promoting the migration of tumour cells along the nerves. In addition, the mediators released by mast cells act on the tumour microenvironment by promoting angiogenesis and vascular leakage, thereby developing a tumour microenvironment that is pro-tumourigenic [26].

### 4.2. Neuroimmune Signalling Pathways

In PDAC tissues with identified PNI, a substance with a significantly elevated concentration was identified: acetylcholine. This neurotransmitter promotes the formation of an immunosuppressive microenvironment through several mechanisms: first, via the epigenetic suppression of CCL5 expression in tumour cells in an HDAC-1-dependent manner, and second, through the activation of nicotinic receptors. Both of these factors caused a reduction in the recruitment of CD8^+^ lymphocytes to the tumour. Furthermore, acetylcholine inhibited IFN-γ production by CD8^+^ cells and mediated the phenotype switch from Th1 to Th2 in a nicotinic receptor-dependent manner, resulting in a decreased Th1/Th2 ratio [18].

When it comes to supporting TME, VIP alters T-lymphocyte responses. As a result, there is a change from Th1-type responses toward Th2-type responses and a reduction in cytotoxic effector-cell activity. This shift involves a decrease in the production of pro-inflammatory cytokines such as IFN-γ. At the same time, it supports the expression of mediators associated with Th2-type responses. Together, these effects facilitate immunological tolerance within the tumour microenvironment. Consequently, VIP-dependent signalling stabilizes an immune-cell phenotype that promotes tumour progression by weakening the antitumour cellular immune response [3].

Throughout the continuous activation of the sympathetic axis, levels of norepinephrine (NE) in plasma and within the tumour are elevated, representing a biologically significant mechanism modulating the antitumour response. Building on this, chronic adrenergic signalling promotes profound immunosuppression by increasing the populations of immunosuppressive cells and inhibiting the effector phenotype of CD8^+^ lymphocytes. As a result, immune cells are markedly impaired in their attempts to eliminate cancer cells, leading to tumour progression [27]. A study conducted by Yang Li et al. showed that higher NE concentrations are linked to improved colonisation of colorectal cancer metastases to the liver due to the interaction with the neuroimmune system. The β2-adrenergic receptors activate the PI3K/Akt pathway, thereby reprogramming the Kupffer cells and increasing the production of CXCL12 in order to create a favourable microenvironment for metastasis. It is believed that stress-induced adrenergic signalling can promote tumour growth and development [28]. Notably, these effects may be reversible with the use of β-blockers, highlighting the key role of β-adrenergic signalling in creating the immunosuppressive microenvironment [27].

### 4.3. PNI, Cold TME, and Resistance to Immunotherapy

Perineural invasion in pancreatic ductal adenocarcinoma is associated with an immunologically ‘cold’ tumour microenvironment that is characterized by a highly immunosuppressive phenotype. Histopathological examination has shown a lack of lymphocytic infiltrates in tumours that exhibit PNI. In contrast, there is a high frequency of neutrophil-like myeloid-derived suppressor cells, especially the CD13hi subset, which have the ability to inhibit T-cell proliferation through the arginase-1 pathway [9].

**Figure 1:**
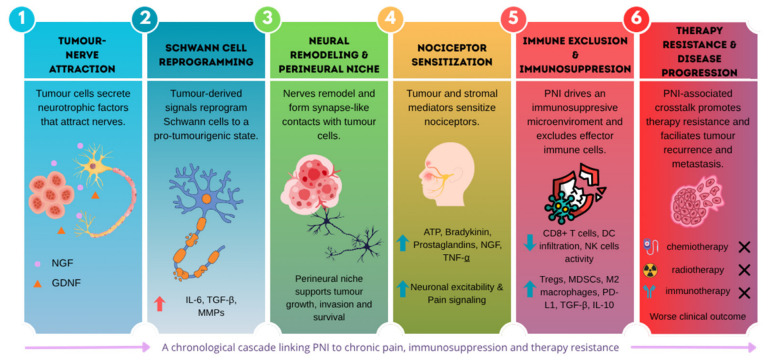
A chronological cascade linking PNI to chronic pain, immunosuppression, and therapy resistance. Vertical arrows indicate a relative increase or decrease in the levels, abundance, expression, or activity of the indicated molecular and cellular factors, as applicable. The “X” symbols indicate reduced efficacy of the corresponding therapeutic approaches. Abbreviations: NGF: nerve growth factor; GDNF: glial cell-line-derived neurotrophic factor; TGF-β: transforming growth factor β; MMPs: matrix metalloproteinases; ATP: adenosine triphosphate; TNF- α: Tumour Necrosis Factor α; DC: dendritic cell; NK: natural killer cell; Tregs: regulatory T cells; MDSCs: myeloid-derived suppressor cells; PD-L1: programmed death-ligand 1.

Cancer-induced nerve injury and perineural invasion have a similar mechanism for the development of resistance to anti-PDL-1 therapy in neurotropic tumours (Figure 1). In cutaneous squamous cell carcinoma, perineural invasion and high levels of neuronal injury markers, including ATF3, have been correlated with non-response to anti-PD-1 therapy. In melanoma, a similar study showed a significant enrichment of the perineural invasion/cancer-induced nerve injury signature in the tumour tissues of anti-PD-1 therapy non-responder patients. This was also shown in metastatic models. In gastric cancer, nerve-injury-associated gene expression profiles were used to distinguish responders to anti-PD-1 therapy from non-responders [29]. Gastric cancer and cSCC commonly have an immune-excluded tumour microenvironment that has been linked to immune suppression and a lack of response to immune checkpoint inhibitors. On the other hand, melanomas are commonly described as having an immunogenic (“hot”) tumour environment; nevertheless, there are certain types of melanomas that develop an immune-excluded tumour environment, especially the ones that are resistant to anti-PD-1 therapy [30,31,32].

### 4.4. Potential Systemic Immunologic Consequences of Local PNI

**Figure 2:**
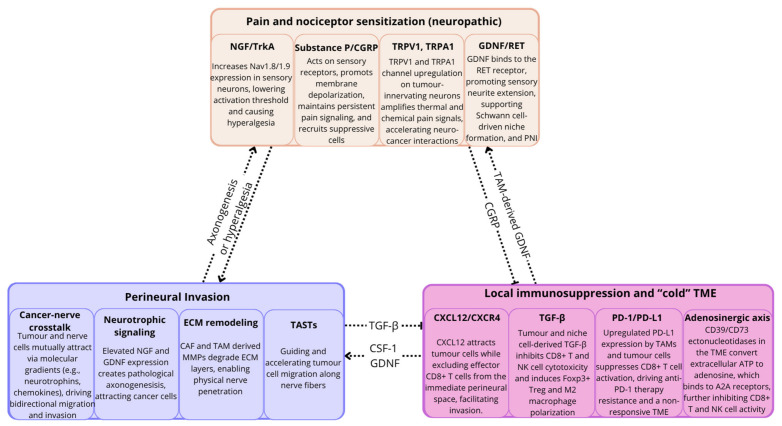
Schematic representation of the tripartite molecular network driving perineural invasion, pain, and local immunosuppression. Abbreviations: NGF: nerve growth factor; TrkA: tropomyosin receptor kinase A; Nav: voltage-gated sodium channels; CGRP: calcitonin gene-related peptide; TRPV1: transient receptor potential vanilloid 1; TRPA1: transient receptor potential ankyrin 1; GDNF: glial cell-line-derived neurotrophic factor; RET: Rearranged during Transfection receptor; TAM: tumour-associated macrophage; CAF: cancer-associated fibroblast; MMPs: matrix metalloproteinases; ECM: extracellular matrix; TASTs: tumour-activated Schwann-cell tracks; TGF-β: transforming growth factor beta; CSF-1: colony-stimulating factor 1; CXCL12: C-X-C motif chemokine ligand 12; CXCR4: C-X-C chemokine receptor type 4; NK cell: natural killer cell; Treg: regulatory T cell; PD-1: programmed cell death protein 1; PD-L1: programmed death-ligand 1; TME: tumour microenvironment; CD39/CD73: ectonucleotidases CD39 and CD73; ATP: adenosine triphosphate; A2A receptors: adenosine A2A receptors.

Perineural invasion in head and neck cancers establishes a specialised neuroimmunological niche. Within this microenvironment, there is enhanced adenosine-axis activity (CD39/CD73–A2A) and the release of neuromodulators, including calcitonin gene-related peptide (CGRP), from nociceptive fibres. CGRP directly and indirectly inhibits cytotoxic CD8^+^ T-cell responses, as well as Th1-type immunity. Concurrently, glial and tumour cells secrete pro-inflammatory mediators such as interleukin-1α, midkine, and TNF-family cytokines. Those molecules modulate the immune-infiltrate composition, promoting suppressive myeloid polarisation. In a study conducted by Li and Xiao, it was highlighted that sustained activation of adenosine pathways and high CD73 expression correlate with poorer responses to systemic anti-PD-1 immunotherapy, suggesting a progression from localised neuroimmune regulation along the nerve to systemic immunological consequences [33]. Figure 2 summarises the key pathways involved in the proposed PNI–pain–immunosuppression triad.

## 5. The PNI–Pain–Immunosuppression Triad Across Organs

**Figure 3:**
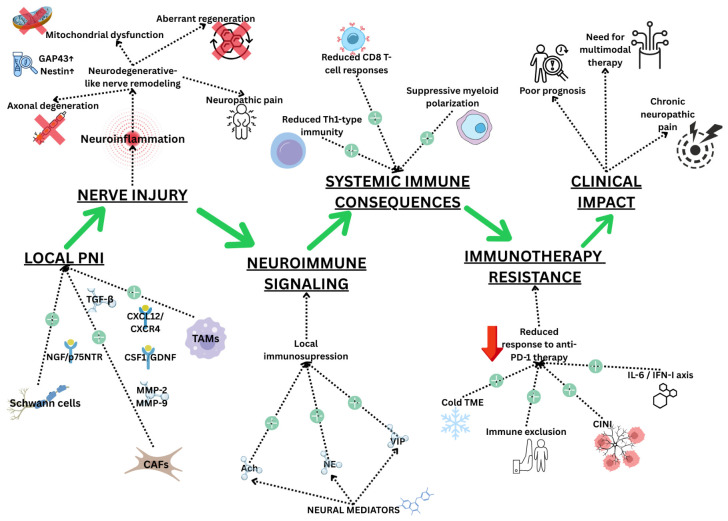
From local perineural invasion to systemic immune consequences and clinical impact. Abbreviations: NGF: nerve growth factor; CXCL12: C-X-C motif chemokine ligand 12; CXCR4: C-X-C chemokine receptor type 4; TGF-β: transforming growth factor β; MMP-2: matrix metalloproteinase-2; MMP-9: matrix metalloproteinase-9; TAMs: tumour-associated macrophages; CAFs: cancer-associated fibroblasts; Ach: acetylcholine; NE: norepinephrine; VIP: vasoactive intestinal peptide; TME: tumour microenvironment; CINI: cancer-induced nerve injury.

The local effect of PNI includes neuroinflammation and neurodegeneration-like remodelling that facilitate the generation of neuroimmune signals and local immunosuppression (Figure 3). The mentioned mechanisms cannot be limited by the local perineural environment and lead to systemic immune abnormalities, which include insufficient antitumour immune response and resistance to immunotherapy. In turn, the mentioned processes may result in unfavourable clinical effects such as poor prognosis and neuropathic pain.

**Figure 4:**
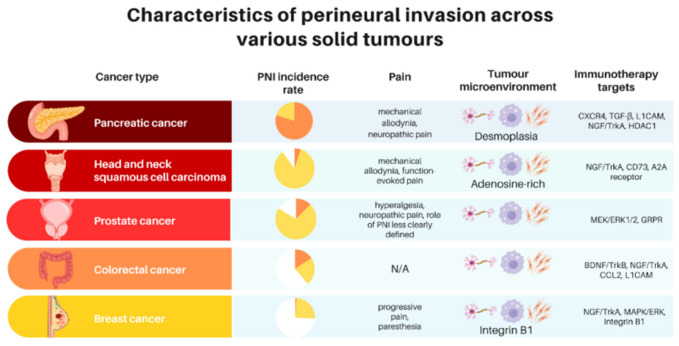
Characteristics of perineural invasion across various solid tumours—a comparative infographic. Abbreviations: C-X-C: chemokine receptor type 4; CXCR4 TGF-β: transforming growth factor β; L1CAM: L1 cell adhesion molecule; HDAC1: histone deacetylase 1; NGF: nerve growth factor; TrkA: tropomyosin receptor kinase A; MEK: mitogen-activated protein kinase; ERK: extracellular signal-regulated kinase; GRPR: gastrin-releasing peptide receptor; BDNF: brain-derived neurotrophic factor; TrkB: tropomyosin receptor kinase B; CCL2: C-C motif chemokine ligand 2; MAPK: mitogen-activated protein kinase.

PNI-positive tumours are characterised by a similar pattern of neuropathic pain, involving hyperalgesia and allodynia. Perineural invasion in HNSCC may manifest clinically as function-evoked pain. The TME shares fundamental components across different malignancies, such as TAMs, CAFs, and Schwann cells. However, PDAC is distinguished by its exceptionally dense desmoplastic stroma. Additionally, PNI in HNSCC is associated with elevated adenosinergic signalling. Numerous immunotherapy targets and corresponding drugs are currently under evaluation. These features of perineural invasion, along with potential immunotherapy targets, are summarised in Table 1 and further visualised in Figure 4.

## 6. Clinical and Therapeutic Implications

### 6.1. PNI as an Independent Prognostic Factor

According to the current AJCC/UICC classifications, perineural invasion usually does not affect the T, N, or M categories, but it remains an important independent prognostic factor [41]. There are some exceptions, such as cutaneous squamous cell carcinoma—in the AJCC 8th classification, perineural invasion of at least 0.1 mm or invasion of a nerve located deeper than the dermis warrants a stage T3 diagnosis. The BWH classification recognises PNI in cSSC as one of the high-risk features [42]. PNI in this cancer was associated with a higher incidence of local recurrence and distant metastasis, as well as a higher risk of death caused by cSCC [43]. In penile cancer, the AJCC 8th edition utilises perineural invasion as a criterion for differentiating between stages T1a (PNI negative) and T1b (PNI positive). Clinically, PNI in this tumour is associated with a higher risk of inguinal lymph-node metastases and higher cancer-specific mortality in comparison with patients without PNI [44].

In head and neck squamous cell carcinomas, such as laryngeal and oral cancers, the presence of PNI correlates with poorer overall survival (OS) and disease-free survival (DFS), as well as increased rates of local and systemic recurrence [45,46].

Studies have shown that PNI is an independent prognostic factor in oesophageal and colorectal cancers. PNI-positive patients with oesophageal squamous cell carcinoma had an increased risk of metastasis and recurrence, as well as worse OS and DFS, but only in pN0 patients. For pN-positive patients, PNI status did not affect OS or DFS [47]. The presence of PNI in patients with colorectal cancer after surgical resection was associated with poorer DFS and OS. At the same time, postoperative chemotherapy was shown to significantly improve outcomes in PNI-positive patients [48].

Similar trends were observed in pancreatic ductal adenocarcinoma. Perineural invasion negatively affected OS in margin-negative (R0) and margin-positive (R1) patients, and its impact on survival was evident, regardless of pN status. Additionally, PNI was the strongest predictor of OS in R0N0 patients [49].

Patients with prostate cancer and perineural invasion after radical prostatectomy had shorter biochemical recurrence-free survival and OS rates than PNI-negative patients, even after adjusting results for p-PSA, T stage, and Gleason score [50].

In breast cancer, PNI had a negative impact on distant metastasis-free survival and disease-specific survival rates. The occurrence of perineural invasion was also associated with higher rates of metastasis to the lymph nodes, invasion of the lymphatic vessels, and a large pathologic tumour size [51].

### 6.2. Diagnostic Imaging of Perineural Invasion

PNI visible in imaging studies is referred to as perineural tumour spread (PNTS) [52]. During the diagnosis of perineural tumour spread, MRI is one of the key tools used for its visualisation. On MRI, PNTS may manifest as nerve expansion with signal intensity comparable to the primary tumour, abnormal contrast enhancement along the course of the affected nerve, and obliteration of the normal perineural fat planes [53,54]. MRI findings in HNSCC may also include widening of the neural foramen [53]. MRI is more effective in imaging larger and more distinct nerves. Smaller nerves (e.g., autonomic nerves surrounding arteries) may remain undetectable, and PNTS detection may then rely on computed tomography (CT) assessment of changes in the perivascular fat signal [54].

In more advanced stages or in post-therapy imaging, fluorodeoxyglucose positron emission tomography (FDG PET) may be helpful in evaluating localisation and the depth of infiltration, typically presenting as linear FDG accumulation along the course of the infiltrated nerve [53,54].

In HNSCC, the detection of PNTS in the primary tumour in imaging studies is always an indication for adjuvant radiotherapy. It is also recommended in cases of extensive microscopic PNI, as well as in aggressive salivary gland cancers containing microscopic PNI [52].

### 6.3. Diagnosis and Management of PNI-Related Pain

PNI-related pain is typically more severe than pain caused by tumours without nerve infiltration and has a substantial negative impact on quality of life and sleep [34,55]. PNI in breast cancer is characterised by progressive pain, paraesthesia, and motor weakness [14]. In HNSCC, it can cause mechanical allodynia and increased function-evoked pain [34,56]. Moreover, higher pretreatment pain intensity has been shown to predict the presence of PNI and is associated with a more advanced disease stage [57].

While surgical resection of the tumour remains the basis of pain treatment, alternative therapeutic methods are required for patients with inoperable tumours or when recurrence appears [23]. To date, no standardised, evidence-based guidelines specifically address the management of PNI-associated pain. Management usually combines pharmacotherapy and interventional methods. This type of pain exhibits features of neuropathic pain [56]. Therefore, agents such as gabapentin, pregabalin, tricyclic antidepressants, or SNRIs may be used, with topical lidocaine considered for allodynia. If needed, combination therapy may be initiated, and opioids are often added due to a mixed pain component [3].

Pain caused by PNI is often resistant to pharmacological management [22,23]. Therefore, radiotherapy is considered in more severe cases. In PDAC, radiotherapy can reduce pain intensity and analgesic requirements [55]. Furthermore, high-intensity focused ultrasound (HIFU) and interventional techniques such as neurolysis or ultrasound-guided plexus blocks have shown efficacy [55,58].

### 6.4. Therapeutic Perspectives in PNI-Positive Tumours

#### 6.4.1. Therapeutic Strategies Targeting Nerve–Tumour Crosstalk

Nerve–tumour crosstalk represents a potential therapeutic target in PNI-positive cancers. Although most interventions remain outside current clinical practice, ongoing studies are exploring potential immunotherapeutic targets, with several candidates already in preclinical or clinical trials [18,33,38,59].

The use of immune checkpoint inhibitors in the treatment of tumours with PNI is often ineffective [29,60]. Baruch et al. examined the mechanism of resistance to PD-1 inhibitors in cSCC, melanoma, and gastric cancer with cancer-induced nerve injury. The authors demonstrated, in mice, that the likely mechanism of this resistance is chronic inflammation associated with PNI and that blocking molecules involved in this process, such as the IL-6 receptor, ATF3, or IFNAR1, restores the effectiveness of PD-1 inhibitor therapy [29]. HDAC1 has also been proposed as a potential target for the inhibition of PDAC-induced immunosuppression, potentially improving CD8^+^ T-cell recruitment, although further research is needed [18]. Bockorny et al. reported that a CXCR4 antagonist, combined with chemotherapy and anti-PD-1 therapy, improved the effectiveness of chemotherapy and prolonged median OS in patients with metastatic PDAC [38]. Another agent being studied to reduce both immunosuppression and fibrosis in murine models is the TGF-β inhibitor, though clinical trials have, so far, failed to demonstrate a significant therapeutic benefit [3,61]. Targeting of adhesion molecules such as L1CAM has been shown to be effective in inhibiting PNI in mouse models [59]. In addition, anti-TrkA and anti-NGF therapies were reported to reduce nerve infiltration in PDAC cells in vitro [3].

In HNSCC, based on the correlation between upregulated adenosinergic activity and immune resistance, it is hypothesised that targeting the adenosinergic pathway through CD73 or A2A receptor blockade could improve the response to anti-PD-1 therapy. The NGF/TrkA axis represents another potential therapeutic target in PNI-positive HNSCC. However, the clinical relevance of these approaches requires further investigation [33].

#### 6.4.2. Modification of the Perineural Immune Niche

Modulation of the tumour microenvironment occurs through interactions with cell populations such as cancer-associated fibroblasts and tumour-associated macrophages. The targeting of CAFs occurs through vitamin D receptor agonists or all-trans retinoic acid, with the aim of inducing them into a quiescent state. Clinical trials are currently investigating the therapeutic potential of this approach [3]. Another strategy is depletion of CAFs using chimeric antigen receptor T cells (CAR-T) or BCL-2 inhibitors. In PDAC mouse models, CAR-T therapy reduced desmoplasia and increased T-cell infiltration [62,63], whereas BCL-2 inhibition enhanced the tumour response to immune checkpoint therapy [64].

Modulation of TAMs includes CSF1R inhibitors to deplete these cells or prevent their recruitment. Alternatively, blocking of the CD47/SIRPα axis can reprogram TAMs from the M2 (protumoural, immunosuppressive) phenotype to the M1 (antitumoural) phenotype. These strategies have demonstrated promising outcomes in preclinical and clinical trials across various malignancies [65]. Furthermore, activation of antitumoural TAMs using CD40 agonists reduced immunosuppression in murine models of PDAC and colorectal cancer [3,66].

#### 6.4.3. Integrating Pain Management and Immunotherapy

Inhibition of the NGF/TrkA axis has also been shown to reduce pain associated with PNI in preclinical studies conducted on mouse models with malignancies such as prostate cancer, breast cancer, sarcoma, and PDAC [3,67]. In a syngeneic rat model of prostate cancer, blocking of the MEK/ERK axis resulted in the elimination of PNI-induced mechanical allodynia [39].

**Figure 5:**
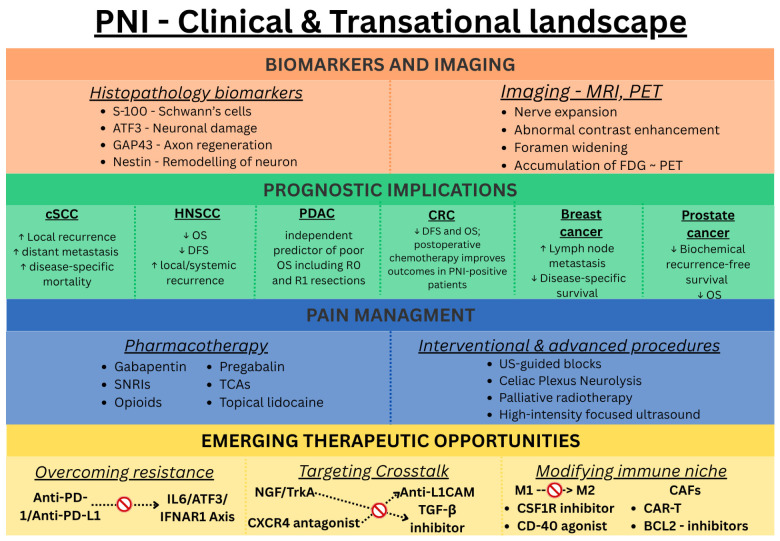
Linking The biology of PNI to clinical practice. Abbreviations: MRI: magnetic resonance imaging; PET: positron emission tomography; ATF3: activating transcription factor 3; FDG: fluorodeoxyglucose; cSCC: cutaneous squamous cell carcinoma; HNSCC: head and neck squamous cell carcinoma; PDAC: pancreatic ductal adenocarcinoma; CRC: colorectal cancer; OS: overall survival; DFS: disease-free survival; SNRIs: serotonin–norepinephrine reuptake inhibitors; TCAs: tricyclic antidepressants; US-guided: ultrasound-guided; PD-L1: programmed death-ligand 1; NGF: nerve growth factor; TrkA: tropomyosin receptor kinase A; CXCR4: C-X-C chemokine receptor type 4; L1CAM: L1 cell adhesion molecule; TGF-β: transforming growth factor β; M1/M2: M1/M2 macrophages; CAFs: cancer-associated fibroblasts; CSF1R: colony-stimulating factor-1 receptor; CAR-T: chimeric antigen receptor T cells.

Despite potential resistance to anti-PD-1 therapy in PNI-positive tumours, a systematic review by Khan et al. demonstrated favourable outcomes in cSCC. The study reported a 61% rate of complete local response or disease stabilisation among treated patients. Additionally, a significant impact in terms of a reduction in neuropathic pain was observed [68,69]. The associations between PNI traits and clinical treatment are summarised in Figure 5.

## 7. Conclusions and Future Directions

As highlighted above, PNI not only facilitates the dissemination of tumour cells but also induces persistent nerve damage, pathological neuroinflammation, and ion-channel remodelling, ultimately leading to chronic neuropathic pain. Perineural invasion represents a significant diagnostic and therapeutic challenge due to the lack of standardised methods for detecting this process, as well as its numerous complications and its detrimental impact on patients’ quality of life. Concurrently, the perineural microenvironment undergoes profound immunosuppression through the recruitment of regulatory T cells (Tregs), M2-polarized tumour-associated macrophages (M2-TAMs), and myeloid-derived suppressor cells (MDSCs), as well as via neuroimmunological signalling mediated by molecules such as acetylcholine (ACh), norepinephrine (NE), and nerve growth factor (NGF), thereby promoting tumour progression and resistance to immunotherapy. PNI contributes to neurodegeneration and local immunosuppression, which, together with pain resulting from nociceptor irritation, may form a functional triad. Based on our current understanding and the reviewed literature, we believe that the proposed triad described above is not universally applicable to all malignancies discussed in this review. Rather, its components appear to be particularly relevant to highly neurotropic malignancies, such as pancreatic carcinoma and head and neck squamous cell carcinoma, although similar mechanisms may also contribute to disease progression in other tumour types reviewed herein. We would like to emphasise that the proposed triad should be regarded as a hypothesis generated from the available evidence rather than a scientifically established concept. Consequently, further experimental and clinical studies are warranted to validate its biological and clinical relevance.

Further investigation of this proposed triad, along with a more comprehensive understanding of the biological processes occurring within the perineural niche, may bring us closer to the development of targeted therapeutic strategies aimed at improving patient outcomes. Moreover, improved insight into the mechanisms underlying neuroinflammation and central sensitisation may help alleviate chronic pain experienced by affected patients.

In our view, future research in this field should focus on a deeper exploration of the perineural niche, as well as on attempts to standardise methods for detecting PNI, including both immunohistochemical and imaging-based approaches. Greater attention should also be devoted to the proposed triad and to strategies aimed at counteracting local immunosuppression, which may help limit or potentially completely stop the progression of the PNI process.

## Figures and Tables

**Table 1:** Characteristics of perineural invasion across various solid tumours.

Cancer Type	PNI Incidence Rate	Characteristics of Pain	Tumour Microenvironment	Immunotherapy Targets
Head and neck squamous cell carcinoma	5.2–90% [4]	Mechanical allodynia and function-evoked pain [34]	Adenosine-rich, neurotrophins, Schwann cells, tumour-associated macrophages, and cancer-associated fibroblasts [11,33]	NGF/TrkA, CD73, and A2A receptor [33]
Pancreatic cancer	80–100% [35,36]	Hyperalgesia and neuropathic pain [37]	Dense desmoplastic stroma, tumour-associated macrophages, cancer-associated fibroblasts, myeloid-derived suppressor cells, regulatory T cells, and Schwann cells [3]	CXCR4, TGF-β, L1CAM, NGF/TrkA, and HDAC1 [3,18,38,39]
Prostate cancer	12.4–83.6% [4]	Hyperalgesia, allodynia, and neuropathic pain; role of PNI in pain generation less clearly defined [12,40]	Neurotrophins, tumour-associated macrophages, cancer-associated fibroblasts, and Schwann cells [11,40]	MEK/ERK1/2 and GRPR [40,41]
Colorectal cancer	15.7–38.9% [4]	NA	Tumour-associated macrophages, cancer-associated fibroblasts, and Schwann cells [7]	BDNF/TrkB, NGF/TrkA, CCL2, L1CAM [7]
Breast cancer	1.14–25.73% [14]	Progressive pain and paresthesia [14]	Extracellular matrix stiffening, fibrosis, tumour-associated macrophages, and integrin B1 [10,14]	NGF/TrkA, MAPK/ERK, and integrin B1 [14]

Abbreviations: NGF: nerve growth factor; TrkA: tropomyosin receptor kinase A; CXCR4: C-X-C chemokine receptor type 4; TGF-β: transforming growth factor β; L1CAM: L1 cell adhesion molecule; HDAC1: histone deacetylase 1; MEK: mitogen-activated protein kinase; ERK: extracellular signal-regulated kinase; GRPR: gastrin-releasing peptide receptor; BDNF: brain-derived neurotrophic factor; TrkB: tropomyosin receptor kinase B; CCL2: C-C motif chemokine ligand 2; MAPK: mitogen-activated protein kinase.

## Data Availability

No new data were created or analysed in this study. Data sharing is not applicable to this article.

## Abbreviations

The following abbreviations are used in this manuscript:

ALS	Amyotrophic lateral sclerosis
ATF-3	Activating transcription factor 3
ATP	Adenosine triphosphate
BDNF	Brain-derived neurotrophic factor
CAFs	Cancer-associated fibroblasts
CGRP	Calcitonin gene-related peptide
CIPN	Chemotherapy-induced peripheral neuropathy
CNS	Central nervous system
CN V	Cranial nerve V
CN VII	Cranial nerve VII
CRC	Colorectal cancer
cSCC	Cutaneous squamous cell carcinoma
CCL2	C-C motif chemokine ligand 2
CSF-1	Colony-stimulating factor-1
CXCL12	C-X-C motif chemokine ligand 12
CXCR4	C-X-C chemokine receptor type 4
DRG	Dorsal root ganglion
DRGs	Dorsal root ganglia
ECM	Extracellular matrix
EMT	Epithelial–mesenchymal transition
ERK	Extracellular signal-regulated kinase
FDG	Fluorodeoxyglucose
GDNF	Glial cell-line-derived neurotrophic factor
GRPR	Gastrin-releasing peptide receptor
HDAC-1	Histone deacetylase 1
HE	Haematoxylin and eosin
HNSCC	Head and neck squamous cell carcinoma
IFN-γ	Interferon gamma
L1CAM	L1 cell adhesion molecule
MAPK	Mitogen-activated protein kinase
MDSC	Myeloid-derived suppressor cell
MDSCs	Myeloid-derived suppressor cells
MEK	Mitogen-activated protein kinase kinase
MMP-2	Matrix metalloproteinase-2
MMP-9	Matrix metalloproteinase-9
Nav	Voltage-gated sodium channels
NE	Norepinephrine
NGF	Nerve growth factor
NGFR/p75NTR	p75 neurotrophin receptor
NK	Natural killer
NK cell	Natural killer cell
NK1	Neurokinin-1
NPY	Neuropeptide Y
OSCC	Oral squamous cell carcinoma
PANR	PDA-associated neural remodelling
PDAC	Pancreatic ductal adenocarcinoma
PD-L1	Programmed death-ligand 1
PNI	Perineural invasion
PNS	Peripheral nervous system
PNTS	Perineural tumour spread
PPSP	Persistent postoperative neuropathic pain
RET	RET receptor complex
RSCs	Reparative Schwann cells
SCC	Squamous cell carcinoma
SCs	Schwann cells
SP	Substance P
TAMs	Tumour-associated macrophages
TASTs	Tumour-activated Schwann-cell tracks
TGF-β	Transforming growth factor β
TME	Tumour microenvironment
TNF- α	Tumour necrosis factor α
Treg	Regulatory T cell
Tregs	Regulatory T cells
Th1	T helper 1
Th2	T helper 2
TrkA	Tropomyosin receptor kinase A
TrkB	Tropomyosin receptor kinase B
TRP	Transient receptor potential
TRPA1	Ankyrin type 1
TRPV1	Vanilloid type 1
VIP	Vasoactive intestinal peptide
